# A Head-to-Head Comparison of the First-Line Treatments for Locally Advanced or Metastatic Urothelial Cancer: Is There Still a Role for Chemotherapy?

**DOI:** 10.3390/cancers16132400

**Published:** 2024-06-29

**Authors:** Lorenzo Gasperoni, Luna Del Bono, Andrea Ossato, Emilio Francesco Giunta, Andrea Messori, Vera Damuzzo

**Affiliations:** 1 Oncological Pharmacy Unit, IRCCS Istituto Romagnolo per lo Studio dei Tumori (IRST) “Dino Amadori”, 47014 Meldola, Italy; lorenzo.gasperoni@irst.emr.it; 2 Azienda Ospedaliera Universitaria Pisana, 56100 Pisa, Italy; lunadelbono@gmail.com; 3 School of Specialization in Hospital Pharmacy, Department of Pharmacy, University of Pisa, 56100 Pisa, Italy; 4 Department of Pharmaceutical and Pharmacological Sciences, University of Padua, 35131 Padova, Italy; andrea.ossato@studenti.unipd.it; 5 Department of Medical Oncology, IRCCS Istituto Romagnolo per lo Studio dei Tumori (IRST) “Dino Amadori”, 47014 Meldola, Italy; 6 HTA Unit, Regional Health Service, 50139 Florence, Italy; 7 Hospital Pharmacy, Vittorio Veneto Hospital, 31029 Vittorio Veneto, Italy; vera.damuzzo@gmail.com; 8 Italian Society of Clinical Pharmacy and Therapeutics (SIFaCT), 10123 Turin, Italy

**Keywords:** indirect comparison, IPDfromKM method, reconstructed individual patient data, overall survival, urothelial cancer, enfortumab vedotin

## Abstract

**Simple Summary:**

Recently, numerous treatments have been approved for patients with previously untreated locally advanced or metastatic urothelial cancer. These combinations prolong survival compared to chemotherapy, which is generally considered the standard of care. Since direct head-to-head comparisons between these innovative treatments are not available, our analysis was aimed at performing indirect comparisons among these agents, including chemotherapy as a common comparator. In handling these indirect comparisons, our study used an innovative evidence-based method (the IPDfromKM technique) that reconstructs individual patient data from published clinical trials. Overall survival was the endpoint of our analysis. Our results showed that enfortumab vedotin plus pembrolizumab was the most effective treatment at levels of statistical significance. The combinations of a PD(L)-1 inhibitor plus chemotherapy ranked second, while monotherapies with a PD(L)-1 inhibitor ranked third. One strength of this analysis is represented by the large number of different treatments that were evaluated and compared to each other.

**Abstract:**

Background: Patients with locally advanced/metastatic urothelial cancer have been conventionally treated with platinum-based chemotherapy. Recently, numerous new treatments have been proposed to improve overall survival (OS) and reduce adverse effects, but no direct head-to-head comparisons among these agents are available. Methods: The treatments evaluated in our analyses included (a) monotherapy with immune checkpoint inhibitors (ICI); (b) combinations of an ICI with chemotherapy; and (c) combinations of an ICI with other drugs. Using OS as the endpoint, a series of indirect comparisons were performed to rank the most effective regimens against both chemotherapy and each other. Our analysis was based on the application of an artificial intelligence software program (IPDfromKM method) that reconstructs individual patient data from the information reported in the graphs of Kaplan–Meier curves. Results: A total of five studies published in six articles were included. In our main analysis, nivolumab plus chemotherapy showed better OS compared to chemotherapy (HR = 0.70, 95% CI: 0.59–0.82), while durvalumab plus tremelimumab showed no OS benefit (HR = 0.95, 95% CI 0.82–1.11). More interestingly, enfortumab vedotin plus pembrolizumab significantly prolonged OS compared to both chemotherapy alone (HR = 0.53, 95% CI 0.45–0.63) and nivolumab plus chemotherapy (HR = 0.76, 95% CI 0.60–0.97). Discussion and conclusion: Among new treatments for locally advanced and metastatic urothelial cancer, enfortumab vedotin plus pembrolizumab showed the best efficacy in terms of OS. Our results support the use of this combination as a first-line treatment in this setting.

## 1. Introduction

The reconstruction of individual patient data from Kaplan–Meier (KM) plots is a novel methodological approach that is increasingly being used to investigate therapeutic questions based on time-to-event endpoints [1,2,3,4], especially when the focus is on the long term. The IPDfromKM method (Individual Patient Data from published Kaplan–Meier survival curves), also known as the “Shiny” method, represents an important new approach to the study of anticancer agents; its computational algorithm has been developed using artificial intelligence [5]. Besides oncology, the IPDfromKM method has been applied to numerous other areas of medical treatments, especially in cardiology [6,7,8]. For these reasons, we chose to use this methodology to conduct an updated analysis of the evidence available for first-line treatment of locally advanced or metastatic urothelial cancer, focusing on clinical trials with long-term follow-up.

In this setting, first-line chemotherapy with platinum plus gemcitabine has been the standard of care for decades. More recently, maintenance treatment with avelumab, an immune checkpoint inhibitor, has been added in patients who respond to platinum, with encouraging results; in fact, in the Javelin-100 trial, avelumab reduced the risk of death by a quarter regardless of PD-L1 expression [9]. However, approximately 50% of patients are ineligible to receive cisplatin [10]; these patients, who typically receive carboplatin plus gemcitabine, have generally a lower survival rate. Recently, several regimens have attempted to address this clinical need in order to improve the outcome of these patients [11].

Although some standard meta-analyses have been performed to compare the efficacy of the main treatment alternatives for this disease (especially immunotherapies [12,13]), long-term survival can be more accurately assessed by relying on the entire KM curves rather than just a hazard ratio. In fact, these KM curves based on reconstructed patients, which contain as many curves as the treatments considered, can provide much more information than that derived from the forest plot typically used in a standard binary meta-analysis. A large number of studies [3,6] have shown that the IPDfromKM method can perform particularly well in these situations.

In our analysis, we used the IPDfromKM methodology to provide a comparative overview of the major treatment options for locally advanced or metastatic urothelial cancer to determine their respective positions in the armamentarium and their comparative effectiveness.

## 2. Materials and Methods

### 2.1. Literature Search and Inclusion Criteria

A systematic search of the PubMed database was conducted to identify randomized controlled trials (RCTs) that met the eligibility criteria for the analysis. The final search was conducted on 22 April 2024. The search term was constructed as follows: [(“urothelial carcinoma” OR “urothelial cancer” OR “bladder carcinoma” OR “bladder cancer”) AND (“immune checkpoint inhibitor” OR ipilimumab OR nivolumab OR pembrolizumab OR atezolizumab OR durvalumab OR cemiplimab OR avelumab)]. The selection of articles was conducted in accordance with the PRISMA algorithm [14].

The main inclusion criteria were (a) phase III trial; (b) first-line treatment for urothelial carcinoma; (c) treatment with ICI in combination or not with other therapies (except tyrosine-kinase inhibitors); (d) overall survival (OS) endpoint; (e) results reported as a KM curve. In order to prevent the duplicate inclusion of the same patients from different trials, we prioritized the most recent publication.

### 2.2. Reconstruction of Individual Patient Data

We reconstructed individual patient data from the KM curves of both the treatment and the control arms of each randomized clinical trial (RCT) using the IPDfromKM approach [3,5]. The KM curves were digitized using Webplotdigitizer (version 4.7 online; https://apps.automeris.io/wpd/, accessed 22 April 2024). Once the digitized curves were determined, the x,y coordinates and the total number of patients and events were entered into the ‘Reconstruct Individual Patient Data’ function of the IPDfromKM software (version: 1.2.3.0 online; last update: 22 March 2022). The software generated the database of reconstructed individual patient data for each RCT arm. These databases included survival time, defined as the difference between the date of enrolment and the last follow-up date, and the endpoint occurrence at the last observation; therefore, patient outcomes were categorized as alive, dead, or censored.

Platinum-based chemotherapy was used as a standard comparison for all other treatments. Pooled control groups were generated by aggregating all patients who received chemotherapy.

### 2.3. Inclusion and Exclusion of Individual Therapeutic Regimens in Our Main Analysis

We considered the following three categories of experimental treatments separately to avoid an excessive number of treatments in the final analysis:

(a) ICI given as monotherapy;
(b) Combinations of ICI with chemotherapy;
(c) Combinations of ICI with other drugs.

An ex ante analysis of comparative efficacy based on indirect comparisons was conducted for each of the three categories mentioned above. Within each of the three categories, this analysis was aimed to select the regimen with the best OS compared to chemotherapy. Thereafter, we included in our main analysis only the regimens that showed a survival benefit in categories (a) and (b). Finally, we compared these regimens of categories (a) and (b) with each of the regimens included in category (c).

### 2.4. Statistical Analysis

The efficacy of the different therapeutic options was evaluated in comparison with that of the pooled control arms by applying the Cox statistic to the endpoint of OS. The results were presented as hazard ratio (HR) with 95% confidence intervals (CIs). To ascertain whether the control groups were homogenous, both the likelihood ratio test and the concordance test were employed according to the statistics of between-trial heterogeneity.

A Cox model and restricted mean survival time (RMST) calculation were employed to perform indirect comparisons between active treatments across all head-to-head combinations. The statistical analyses were conducted using the “survival” package in the R platform (version 4.3.2).

## 3. Results

### 3.1. Trial Selection and Design of Analysis

**Figure 1:**
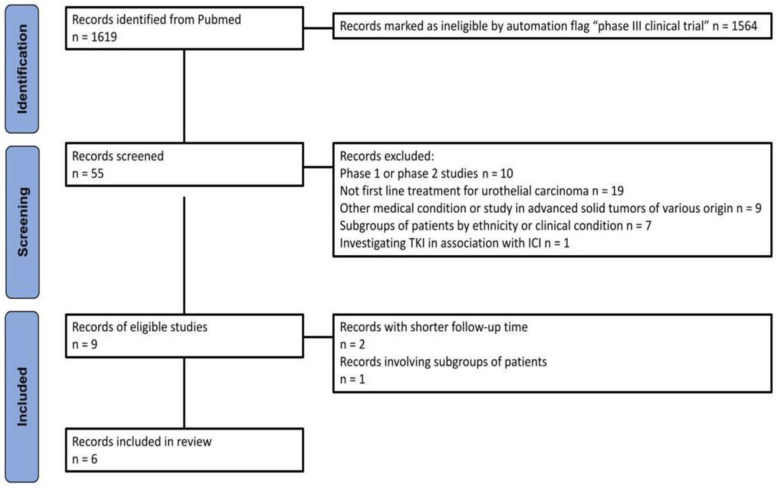
The PRISMA flowchart illustrates the process of trial selection. Abbreviations: TKI, tyrosine kinase inhibitor.

Figure 1 outlines the methodology, based on the PRISMA algorithm, employed for the selection of included trials. In particular, our initial search identified 1619 records; of these, 55 randomized interventional clinical trials were then selected using the PubMed filter. After the application of inclusion criteria, a total of five trials, corresponding to six publications, were included in our analysis (Table 1 and Table 2). These trials investigated the efficacy of ICI as monotherapy or in combination with chemotherapy or other drugs, with gemcitabine plus platinum-based regimen serving as the common comparator.

In the initial phase of our analyses, we reconstructed individual patient data from the original KM curves of the patient cohorts listed in Table 2. We then proceeded to assess OS in the following groups (as previously described in Section 2.3):

(a) OS of ICIs used as monotherapy vs. standard chemotherapy;
(b) OS of ICIs combined with chemotherapy vs. standard chemotherapy;
(c) OS of ICI combined with other drugs vs. standard chemotherapy.

Before assessing the OS data for the above three groups, the reconstructed patients deriving from all the control arms were pooled together to create a common comparator, consisting of patients receiving platinum-based chemotherapy plus gemcitabine. A preliminary heterogeneity analysis was conducted to study these control arms: since no significant heterogeneity was found, this allowed us to merge the patients of these control arms into a single control group, which was used as a common comparator.

### 3.2. Ex Ante Analysis: Regimens’ Selection Based on Overall Survival

**Figure 2:**
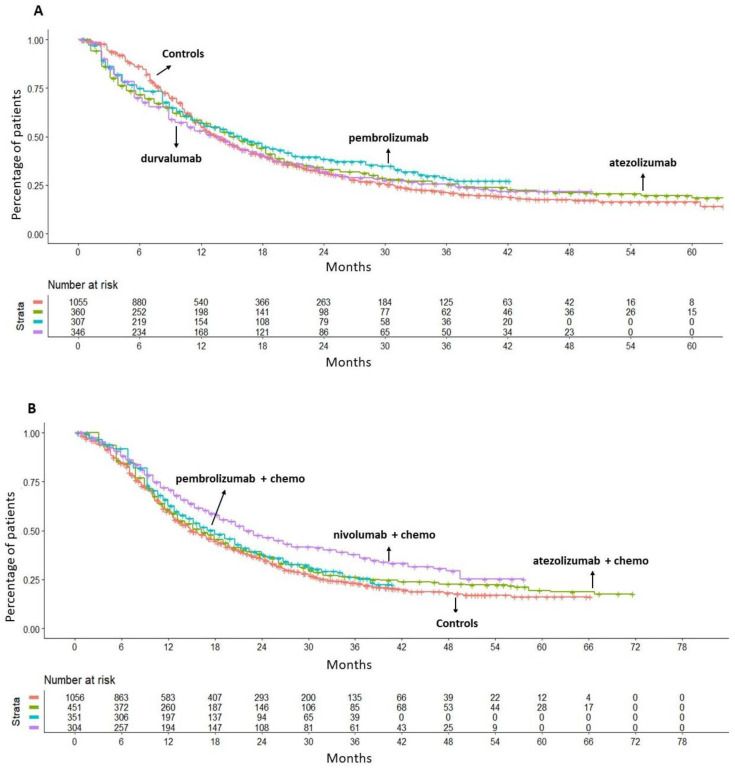
(**A**) IPDfromKM method applied to control arms (n = 1055 from three trials; in red) and three ICI treatments: (a) atezolizumab 1200 mg (n = 360; in green); (b) pembrolizumab 200 mg (n = 307; in light blue); and (c) durvalumab 1500 mg (n = 346; in purple). (**B**) Kaplan–Meier curves generated after reconstructing patient-level data from three control arms (n = 1056; in red) and three ICI plus chemotherapy (gemcitabine and platinum-based chemotherapy) regimens: (a) atezolizumab plus chemotherapy (n = 451; in green); (b) pembrolizumab plus chemotherapy (n = 351; in light blue); and (c) nivolumab plus chemotherapy (n = 304; in purple). Endpoint: overall survival (OS), time in months.

In the comparison between monotherapy with an ICI (atezolizumab, pembrolizumab, or durvalumab) vs. gemcitabine plus platinum-based chemotherapy, it was found that ICI as monotherapy did not produce any significant OS benefit; the values of HR were as follows: atezolizumab, HR = 0.98 (95% CI 0.85–1.13); pembrolizumab, HR = 0.89 (95% CI 0.76–1.05); durvalumab, HR = 1.03 (95% CI 0.89–1.19) (Figure 2A). Owing to the lack of a survival benefit, none of these three regimens was eventually included in our final analysis. Quite interestingly, no significant heterogeneity was found between the groups treated with chemotherapy in Imvigor-130, Keynote-361, and CheckMate-901 studies, which displayed a similar pattern of OS. This finding confirmed the rationale for our decision to merge the three control groups into a single control group, which was used as a common comparator (Figure S1A in the Supplementary Material).

When ICI plus chemotherapy regimens were compared with this common comparator, neither atezolizumab plus chemotherapy (HR = 0.91, 95% CI 0.80–1.04) nor pembrolizumab plus chemotherapy (HR = 0.89, 95% CI 0.76–1.03) were superior to chemotherapy alone in terms of OS. Conversely, nivolumab plus chemotherapy demonstrated a significant survival advantage in comparison to chemotherapy (HR = 0.70, 95% CI 0.59–0.82) with a reduction of 30% in the death risk at a median follow-up of 33.6 months (Figure 2B).

### 3.3. Main Analysis: Overall Survival of Chemotherapy-Free Regimens Compared with ICI Plus Chemotherapy

In light of the results presented in previous sections, our main analysis was conducted to assess the efficacy of ICI combined with chemotherapy-free treatments compared to the only regimen of ICI plus chemotherapy (nivolumab plus chemotherapy) that proved to be superior to chemotherapy alone in terms of OS. Therefore, the following three treatments were evaluated: (i) nivolumab plus chemotherapy; (ii) durvalumab plus tremelimumab; and (iii) enfortumab vedotin plus pembrolizumab.

A preliminary heterogeneity analysis (Figure S1B) involved the control arms of three trials: CheckMate-901, DANUBE, and EV-302. As shown in Figure S1B, the KM curve of the CheckMate-901 control arm (study by van der Heiden et al., 2023 [20]) suggests that the controls in this study survived longer than the controls of both the DANUBE (study by Powles et al., 2020 [19]) and EV-302 (study by Powles et al., 2024 [18]) studies. This might suggest that the patients enrolled in the Check-Mate-901 trial had more favorable characteristics.

**Figure 3:**
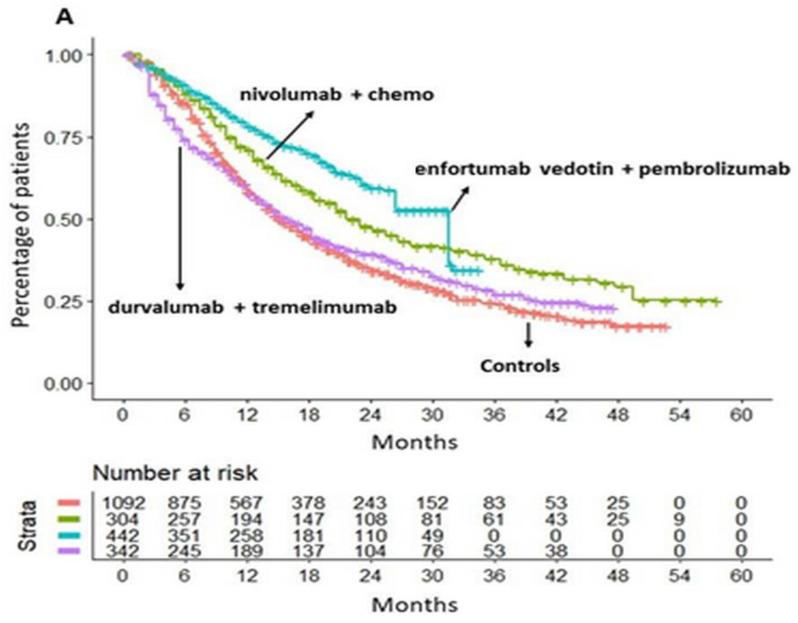

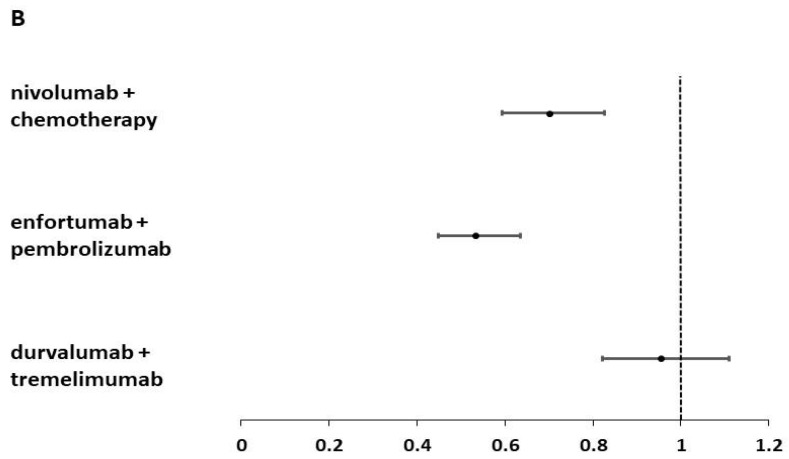
(**A**) IPDfromKM method applied to control arms (n = 1092 from three trials; in red) and three different treatments: (a) nivolumab plus chemotherapy (n = 304; in green); (b) enfortumab vedotin plus pembrolizumab (n = 442; in light blue); and (c) durvalumab plus tremelimumab (n = 342; in purple). (**B**) Forest plot showing the overall survival of first-line treatment options for urothelial cancer patients treated with the best three treatments. Values are reported as HR of overall survival compared with controls.

Our main analysis is shown in Figure 3 (panels A and B). Its results indicate that nivolumab in combination with chemotherapy demonstrated superior efficacy in terms of OS compared to chemotherapy alone (HR = 0.70, 95% CI: 0.59–0.82). In contrast, durvalumab in combination with tremelimumab did not demonstrate any OS benefit compared to the aforementioned controls (HR = 0.95, 95% CI 0.82–1.11).

A noteworthy result of our main analysis is that the combination of enfortumab vedotin plus pembrolizumab demonstrated a significantly superior OS compared to chemotherapy alone (HR = 0.53, 95% CI 0.45–0.63) and also to nivolumab plus chemotherapy (HR = 0.76, 95% CI 0.60–0.97). The magnitude of the survival benefit was considerable in both cases. In Figure 3A, the multi-treatment KM curves based on reconstructed patients provide an effective graphical synthesis of all results generated by our main analysis, thus confirming the advantage of the IPDfromKM method in communicating this type of results. Figure 3B shows the forest plot of HRs with 95% CI for the three therapeutic approaches selected for this analysis in comparison with the pooled control groups given chemotherapy.

We calculated the RMST at 34 months to obtain a more comprehensive understanding of the survival benefit produced by enfortumab vedotin plus pembrolizumab over nivolumab plus chemotherapy. It was found that the combination of nivolumab plus chemotherapy provided a survival advantage of 3.5 months in comparison to chemotherapy alone (RMST = 21.78 months; 95% CI 20.40–23.15 vs. RMST= 18.88; 95% CI 17.6–19.06, respectively). The combination of enfortumab vedotin plus pembrolizumab was found to provide a survival advantage of two additional months (RMST = 24.12, 95% CI 22.92–25.31) in comparison to nivolumab plus chemotherapy. In summary, the combination of enfortumab vedotin and pembrolizumab seems to be the optimal therapeutic option for the treatment of locally advanced or metastatic urothelial cancer.

## 4. Discussion

This study provides an updated review of the current evidence for the first-line treatment of locally advanced and metastatic urothelial cancer using the IPDfromKM method. Our systematic approach and its typical presentation of survival outcomes make it clear that enfortumab vedotin plus pembrolizumab is the superior option, not only over standard chemotherapy, as shown in the pivotal trial [18], but also over all other treatment options. The endpoint of our analysis was OS, which provides strong confidence in the results of our analysis.

While the heterogeneity of survival in the control arms might have overestimated the effect of nivolumab plus chemotherapy, the significance of the additional benefit seen with enfortumab vedotin plus pembrolizumab is clear, and indeed this regimen has quickly emerged as the new standard of care for this disease. The benefit of this combination is independent of PD-L1 expression (consistent with previous reports for pembrolizumab in the first-line setting) but also independent of clinical factors such as Eastern Cooperative Oncology Group Performance Status (ECOG-PS) or the presence of liver metastases [17,18].

The advantage of the IPDfromKm method is that all patients studied in included trials are firstly reconstructed from the analysis of KM curves; then, those patients who are treated with the same regimen are pooled together through an artificial intelligence algorithm. In this way, the typical final graph of a IPDfromKM analysis presents as many KM curves as the number of treatments being compared, so these multi-treatment KM curves based on reconstructed patients can easily be used to perform any combinations of indirect head-to-head comparisons.

Apart from KM curves, forest plots are typical tools that are generally used to present survival outcomes in the context of a binary meta-analysis. The advantage of the IPDfromKM method is that the times at which individual events occur are accurately taken into account (unlike in a binary meta-analysis, where the forest plot by definition loses this piece of information). Another advantage of a purely communicative nature is that the KM curves of the various treatments estimated from reconstructed patients (as those reported in the figure of our main analysis, Figure 3A) offer a simple but effective way to communicate the results of the IPDfromKM analysis. The main disadvantage of the IPDfromKM method is that survival analyses based on patient subgroups can only be performed if the Kaplan–Meier curve for the specific subgroup is available in the published study.

The editorial by Niegisch et al. accompanying the publication of the enfortumab study in The New England Journal of Medicine [21] noted that while the increased efficacy seen with this new combination is undeniable, the question of cost arises. In the US, Ike et al. [22] calculated that the estimated annual cost of treatment with enfortumab vedotin plus pembrolizumab was 3.8 times higher than the cost of platinum-based chemotherapy followed by maintenance avelumab (USD 455,630 vs. USD 120,253 per patient, respectively), which is the reference treatment for pricing in this context. Finally, it should be stressed that the results of the Javelin Bladder 100 trial are likely considerably influenced by a selection bias owing to the inclusion of only platinum-eligible responders; this justifies the exclusion of this study from our comparative analysis.

As mentioned above, our preliminary heterogeneity analysis (Figure S1B) indicates that patients in the CheckMate-901 control arm lived longer than patients in the DANUBE and EV-302 control arms. The probable selection bias in CheckMate-901 can be explained because patients had to be eligible for cisplatin therapy, without the treatment alternative of carboplatin, yielding a population of more fit patients with better survival probabilities. However, this would further strengthen the superiority of enfortumab vedotin plus pembrolizumab according to the results of this head-to-head comparison. It is important to emphasize that, in the near future, the platinum eligibility criteria will be less and less used in clinical practice after the widespread adoption of enfortumab vedotin plus pembrolizumab in the first-line setting, and this will likely influence also the choice of the subsequent lines of therapy. Regarding adverse events with the drug combinations, in the EV-302 trial, the percentage of grade 3–4 adverse events favored the experimental arm (55.9% versus 69.5% in the control arm) [18]. In contrast, in the CheckMate-901 trial, the percentage of grade 3–4 adverse events favored the control arm (51.7% versus 61.8% in the experimental arm) [20]. In the DANUBE trial, a higher rate of discontinuation was observed in the durvalumab plus tremelimumab arm compared to the chemotherapy arm (24% vs. 17%), although grade ≥ 3 adverse events were significantly less frequent in the immunotherapy arm compared to the control arm (27.9 vs. 60.3%) [19]. However, to better interpret these findings, the different profiles and frequencies of the individual adverse events should be taken into account. While enfortumab vedotin has a lower percentage of grade 3–4 adverse events compared to platinum-based chemotherapy, it is important to note that peripheral sensory neuropathy associated with enfortumab vedotin could have a substantial impact [23,24], particularly given the expected high number of cycles of this drug when administered in the first-line setting. Long-term safety data and patient-reported outcomes are needed to clarify this issue [25,26]. Apart from the well-known higher rate of enfortumab-related peripheral sensory neuropathy, diarrhea and alopecia were also more frequent in the EV-302 experimental arm, which might have a significant impact on quality of life [18]. Taken together, these data suggest that clinicians should consider the safety profile when selecting first-line therapy for advanced or metastatic urothelial cancer.

## 5. Conclusions

This study used an innovative evidence-based technique to reconstruct individual patient data from published clinical trials focusing on first-line treatments for locally advanced or metastatic urothelial cancer. The aim of our analysis was twofold: Firstly, we sought to provide an updated overview of the main first-line treatments available for advanced urothelial cancer, highlighting the significant increase in efficacy seen with the latest regimen based on enfortumab vedotin, published just a few months ago in 2024. On the other hand, this study provides another important confirmation of the excellent performance of the IPDfromKM method in generating an original analysis based on indirect comparisons. Taking into account the inherent limitations of this technique, our results confirm that enfortumab vedotin plus pembrolizumab is the most effective combination in treatment-naive patients in terms of survival benefit; however, other aspects—namely cost and safety—should be further investigated in order to draw sound conclusions about the comparison between these treatments and to better assess the overall benefit of enfortumab plus pembrolizumab given as first-line therapy.

## Figures and Tables

**Table 1:** Patients’ characteristics in included trials.

Trial (First Author, Year of Publication)	Treatments Arms	Number of Patients	Median Age	ECOG 0-1	Upper Tract Primary Tumor Site (%)	Liver Metastases (n; %)	Lymph Node Only Metastases (n; %)
IMvigorl30 combo (Grande, 2024 [15])	Exp: Atezolizumab + Platinum + Gemcitabine	451	69	86%	17%	95; 21%	80; 18%
	C: Placebo + Platinum + Gemcitabine	400	67	90%	26%	91; 23%	67; 17%
IMvigorl30 mono (Bamias, 2024 [16])	Exp: Atezolizumab	360	67	91%	25%	85; 24%	70; 19%
	C: Placebo + Platinum + Gemcitabine	359	67	90%	26%	84; 23%	ND
KeyNote-361 (Powles, 2021 [17])	Exp1: Pembrolizumab +Platinum + Gemcitabine C: Platinum + Gemcitabine	351 352	69 69	94% 94%	18% 23%	78; 22% 74; 21%	81; 23% 94; 27%
	Exp2: Pembrolizumab C: Platinum + Gemcitabine	307 352	68 69	92% 94%	21% 23%	65; 21% 74; 21%	64; 21% 94; 27%
EV-302 (Powles, 2024 [18])	Exp: Enfortumab vedotin + Pembrolizumab	442	69	97%	30%	100; 23%	103; 23%
	C: Platinum + Gemcitabine	444	69	97%	23%	99; 22%	104; 23%
DANUBE (Powles, 2020 [19])	Exp1: Durvalumab + Tremelimumab C: Platinum + Gemcitabine	342 344	68 68	100% 100%	22% 25%	80; 23% 93; 27%	73; 21% 77; 22%
	Exp2: Durvalumab C: Platinum + Gemcitabine	346 344	67 68	100% 100%	18% 25%	ND 93; 27%	61; 18% 77; 22%
CheckMate-901 (van der Heijden, 2023 [20])	Exp: Nivolumab + Cisplatin + Gemcitabine	304	65	99%	ND	64; 21%	ND
	C: Placebo + Cisplatin + Gemcitabine	304	65	100%	ND	64; 21%	ND

**Table 2:** Treatments and survival information of included trials.

Trial (First Author, Year of Publication]	Treatments Arms	Number of Patients	Median Follow-Up	Median OS (mo)	PDL-1 Expression
IMvigorl30 combo (Grande, 2024 [15])	Exp: Atezolizumab + Platinum + Gemcitabine	451	13.4 mo	Exp: 16.1	24% IC2/3
	C: Placebo + Platinum + Gemcitabine	400		C: 13.4 HR 0.85 (95% CI 0.73–1.00) *p* = 0.023	(PD-L1 immune cell expression status (IC0 [<1%] vs. IC1 [≥1% and <5%] vs. IC2/3 [≥5%]) ^1^
IMvigorl30 mono (Bamias, 2024 [16])	Exp: Atezolizumab	360	13.4 mo	Exp: 15.2	24% IC2/3 ^2^
	C: Placebo + Platinum + Gemcitabine	359		C: 13.3 HR 0.95 (95% CI 0.80–1.12) *p* = 0.023	(PD-L1 immune cell expression status (IC0 [<1%] vs. IC1 [≥1% and <5%] vs. IC2/3 [≥5%])
KeyNote-361 (Powles, 2021 [17])	Exp1: Pembrolizumab +Platinum + Gemcitabine C: Platinum + Gemcitabine	351 352	31.7 mo	Exp1: 17.0 C: 14.3 HR 0.86 (95% CI 0.72–1.02) *p* = 0.0407	47% PD-L1 High CPS ≥ 10 ^3^
	Exp2: Pembrolizumab C: Platinum + Gemcitabine	307 352	31.7 mo	Exp2: 15.6 C: 14.3 HR 0.92 (95% CI 0.77–1.11)	
EV-302 (Powles, 2024 [18])	Exp: Enfortumab vedotin + Pembrolizumab	442	17.2 mo	Exp: 31.5	57% PD-L1 High CPS ≥ 10 ^4^
	C: Platinum + Gemcitabine	444		C: 16.1 HR 0.47 (95% CI 0.38–0.58) *p* = < 0.001	
DANUBE (Powles, 2020 [19])	Exp1: Durvalumab + Tremelimumab C: Platinum + Gemcitabine	342 344	41.2 mo	Exp1: 15.1 C: 12.1 HR 0.85 (95% CI 0.72– 1.02) *p* = 0.075	60% PD-L1 High ^5^
	Exp2: Durvalumab C: Platinum + Gemcitabine	346 344		Exp2: 14.4 C: 12.1 HR 0.89 (95% CI 0.71– 1.11) *p* = 0.30	
CheckMate-901 (van der Heijden, 2023 [20])	Exp: Nivolumab + Cisplatin + Gemcitabine	304	33.6 mo	Exp: 21.7	36% ≥1% ^6^
	C: Placebo + Cisplatin + Gemcitabine	304		C: 18.9 HR 0.78 (95% CI 0.63– 0.96) *p* = 0.02	

^1,2^ Testing of PD-L1 IC expression on ICs as a proportion of the tumor area (SP142 PD-L1 immunohistochemical assay; Ventana Medical Systems. ^3^ PD-L1 status was assessed using the PD-L1 IHC 22C3 pharmDx assay and measured using the CPS, defined as the number of PD-L1-staining cells (tumor cells, lymphocytes, and macrophages) divided by the total number of viable tumor cells, multiplied by 100. ^4^ Programmed death ligand 1 (PD-L1) expression was assessed with the use of the PD-L1 IHC 22C3 pharmDx assay (Agilent Technologies). The combined positive score (CPS) was defined as the total number of programmed death ligand 1 (PD-L1)-staining cells (tumor and immune cells, lymphocytes, and macrophages) divided by the total number of viable tumor cells, multiplied by 100. ^5^ High PD-L1 expression was defined as at least 25% of tumor cells with membrane staining or at least 25% of immune cells staining for PD-L1 at any intensity if more than 1% of the tumor area contained immune cells, or 100% of immune cells staining for PD-L1 at any intensity if 1% of the tumor area contained immune cells. ^6^ PD-L1 status was defined according to the percentage of positive staining of tumor-cell membrane (minimum, 100 tumor cells) that could be evaluated with the use of an immunohistochemical assay for PD-L1. Abbreviations: Exp, experimental; C, controls; IC2/3, assay for expressing PD(L)-1 positivity.

## Data Availability

The data presented in this study are available in the article and Supplementary Material.

## Supplementary Materials

The following supporting information can be downloaded at: https://www.mdpi.com/article/10.3390/cancers16132400/s1, Figure S1. Kaplan-Meier curves generated from reconstruction of individual patient data from control arms of included trials. Panel A: Grande et al., 2024 (n = 400; in red [15]); Powles et al., 2021 (n = 352; in green [17]); van der Heijden et al., 2023 (n = 304; in blue [20]). Panel B: Powles et al., 2024 (n = 444; in red [18]); Powles et al., 2020 (n = 344; in green [19]) and van der Heijden et al., 2023 (n = 304; in blue [20]). N: number of enrolled patients. Endpoint: overall survival (OS), Time in months. See the reference list at the end of this file.
