# Supplementary material for: A Head-to-Head Comparison of the First-Line Treatments for Locally Advanced or Metastatic Urothelial Cancer: Is There Still a Role for Chemotherapy?
Source: Cancers (Basel). 2024 Jun 29;16(13):2400. doi: 10.3390/cancers16132400 (PMC11240305; doi:10.3390/cancers16132400)

Supplementary Material

A Head-to-Head Comparison of The First-Line Treatments for Locally Advanced or Metastatic Urothelial Cancer: Is There Still a Role for Chemotherapy?

Lorenzo Gasperoni [1§], Luna Del Bono [2,3§], Andrea Ossato [4,8], Emilio Francesco Giunta[5], Andrea Messori [6§,*] and Vera Damuzzo[7,8§]

[1] Oncological Pharmacy Unit, IRCCS Istituto Romagnolo per lo Studio dei Tumori (IRST) "Dino Amadori", 47014 Meldola, Italy.

[2] Azienda Ospedaliera Universitaria Pisana, 56100 Pisa, Italy;

[3] School of Specialization in Hospital Pharmacy, Department of Pharmacy, University of Pisa, Pisa, Italy.

[4] Department of Pharmaceutical and Pharmacological Sciences, University of Padua, 35131 Padova, Italy

[5] Department of Medical Oncology, IRCCS Istituto Romagnolo per lo Studio dei Tumori (IRST) 'Dino Amadori', Meldola, Italy;

[6] HTA Unit, Regional Health Service, 50139 Florence, Italy;

[7] Hospital Pharmacy, Vittorio Veneto Hospital, 31029 Vittorio Veneto, Italy;

[8.] Italian Society of Clinical Pharmacy and Therapeutics (SIFaCT), 10123 Turin, Italy.

* Correspondence: andrea.messori.it@gmail.com

§ These Authors contributed equally to the work.

Figure S1. Kaplan-Meier curves generated from reconstruction of individual patient data from control arms of included trials. **Panel A**: Grande et al., 2024 (n=400; in red [15]); Powles et al., 2021 (n=352; in green[17]); van der Heijden et al., 2023 (n=304; in blue [20]). **Panel B**: Powles et al., 2024 (n=444; in red [18]); Powles et al., 2020 (n=344; in green [19]) and van der Heijden et al., 2023 (n=304; in blue [20]). N: number of enrolled patients. Endpoint: overall survival (OS), Time in months. See the reference list at the end of this file.

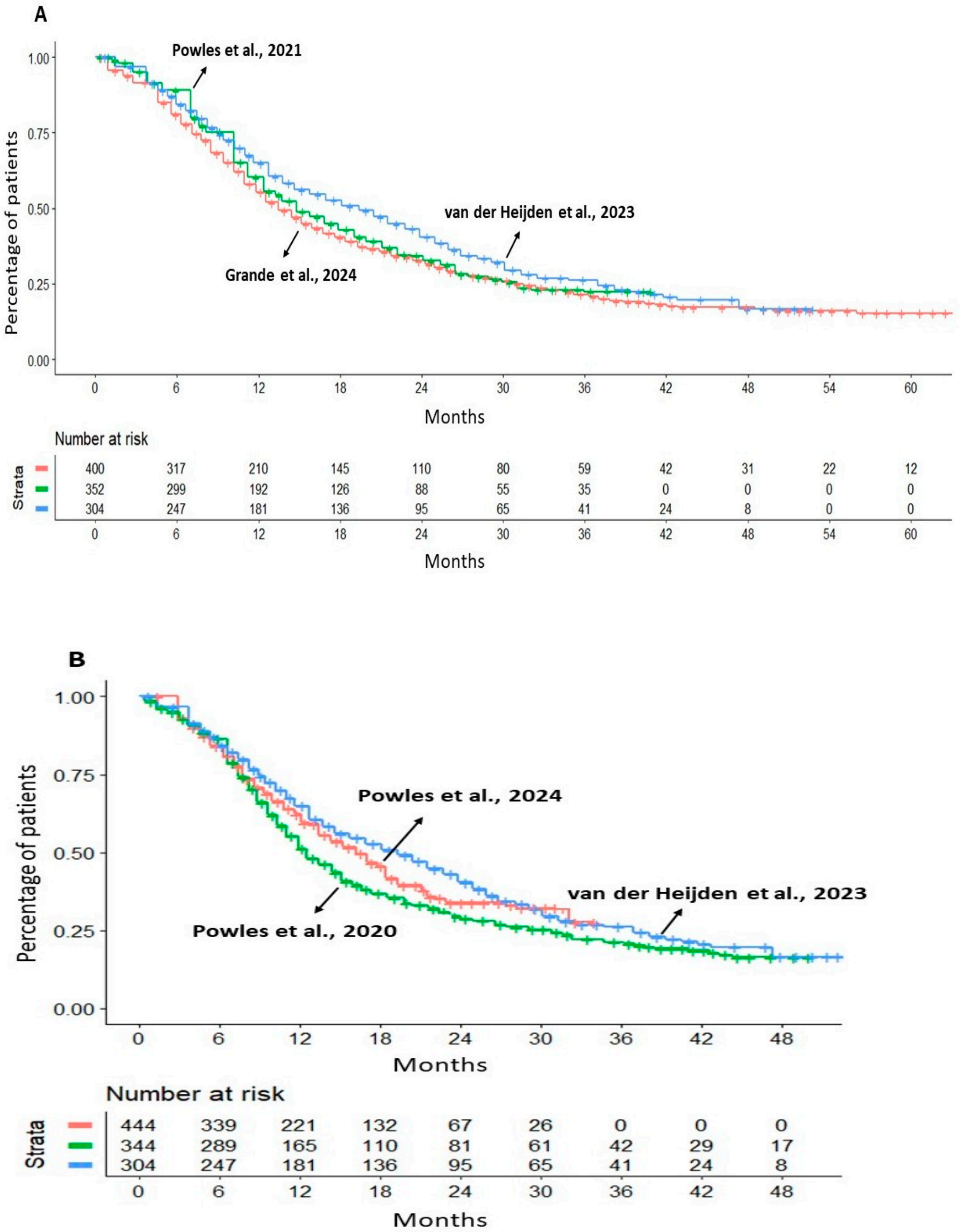

Supplement: Supplementary file 1 [file cancers-16-02400-s001.zip › cancers-3059824-supplementary.pdf]
